# Comparative Phosphoproteomic Analysis of Barley Embryos with Different Dormancy during Imbibition

**DOI:** 10.3390/ijms20020451

**Published:** 2019-01-21

**Authors:** Shinnosuke Ishikawa, José Barrero, Fuminori Takahashi, Scott Peck, Frank Gubler, Kazuo Shinozaki, Taishi Umezawa

**Affiliations:** 1 Graduate School of Bio-Applications and Systems Engineering, Tokyo University of Agriculture and Technology, Koganei, Tokyo 184-8588, Japan; s177676w@st.go.tuat.ac.jp; 2 CSIRO Agriculture and Food, Canberra ACT 2601, Australia; jose.barrero@csiro.au (J.B.); frank.gubler@csiro.au (F.G.); 3 Gene Discovery Research Group, RIKEN Center for Sustainable Resource Science, Tsukuba, Ibaraki 305-0074, Japan; fuminori.takahashi@riken.jp (F.T.); kazuo.shinozaki@riken.jp (K.S.); 4 Department of Biochemistry, University of Missouri, Columbia, MO 65211, USA; pecks@missouri.edu; 5 Faculty of Agriculture, Tokyo University of Agriculture and Technology, Fuchu, Tokyo 183-8538, Japan; 6 PRESTO, Japan Science and Technology Agency, Kawaguchi, Saitama 332-0012, Japan

**Keywords:** phosphoproteome, barley, seed dormancy, germination, imbibition, after-ripening

## Abstract

Dormancy is the mechanism that allows seeds to become temporally quiescent in order to select the right time and place to germinate. Like in other species, in barley, grain dormancy is gradually reduced during after-ripening. Phosphosignaling networks in barley grains were investigated by a large-scale analysis of phosphoproteins to examine potential changes in response pathways to after-ripening. We used freshly harvested (FH) and after-ripened (AR) barley grains which showed different dormancy levels. The LC-MS/MS analysis identified 2346 phosphopeptides in barley embryos, with 269 and 97 of them being up- or downregulated during imbibition, respectively. A number of phosphopeptides were differentially regulated between FH and AR samples, suggesting that phosphoproteomic profiles were quite different between FH and AR grains. Motif analysis suggested multiple protein kinases including SnRK2 and MAPK could be involved in such a difference between FH and AR samples. Taken together, our results revealed phosphosignaling pathways in barley grains during the water imbibition process.

## 1. Introduction

The switch from dormancy to germination is one of important transition steps in the life cycle of plants, because it will be the first and most fundamental factor determining their survivability. During the evolutionary process, seeds have evolved to germinate only in favorable seasons or places and dormancy is the mechanism that inhibits germination [1]. Seed dormancy is a complex trait regulated by many genetic and environmental factors [2,3,4,5], and during plant domestication, the dormancy and germination behavior of different species are set to fit their purpose. Most of domesticated cereals have been selected for uniform and synchronized germination by selection for weakened seed dormancy, which collaterally has made them prone to suffer pre-harvested sprouting (PHS) when moist conditions appear at harvesting seasons [6]. Understanding the mechanisms that operate during dormancy release will be very important to design molecular strategies to reinforce dormancy and provide protection against PHS. Barley is a good model to study seed dormancy regulation in cereals because freshly-harvested barley grains retain relatively high levels of dormancy [7].

To study seed dormancy, we can dissect this trait into three stages: acquisition, maintenance and decay [8,9]. It is well known that the phytohormone abscisic acid (ABA) has a critical role in all stages. During seed maturation, ABA accumulates and imposes the temporal quiescent state known as dormancy. After imbibition, the dormant seed (freshly harvested; FH) will be able to maintain high levels of ABA, thus blocking germination: the quiescent dry seed rapidly resumes metabolic activity, and ABA represses embryo growth (embryo-based dormancy). On the other hand, the seed husk physically inhibits oxygen absorption, and also constrains embryo growth (coat-based dormancy). However, in the non-dormant seed (after-ripened; AR), the ABA content is reduced during imbibition and the signaling repressed, which allows the germination to occur: gibberellic acid-pathways are activated, cell walls are weakened, embryo grows and finally coleorhiza appears through the husk-completing germination [1].

To understand the germination process, previous studies have performed a large-scale gene expression analyses of FH and AR seeds in Arabidopsis or barley during imbibition [10,11,12,13,14,15,16]. These studies revealed the differences in transcriptome between both states. In addition to transcriptional regulation, it has been reported that post-translational modifications (PTM), including phosphorylation, S-nitrosylation, carbonylation, glycosylation and oxidation, have a role in the regulation of seed dormancy and germination [17,18,19,20]. Among them, protein phosphorylation is fundamentally involved in the core ABA signaling pathway [21,22,23]. Furthermore, a protein kinase, MKK3, has been recently identified as a major quantitative trait locus (QTL) for grain dormancy in both barley and wheat [24,25]. Although these results indicate the importance of protein phosphorylation in seed dormancy and germination, the elements of the phosphosignaling pathways in cereal grains are still unsolved.

Taking advantage of the barley model system using FH and AR grains with contrasting dormancy levels, we have performed a large-scale phosphoproteomic analysis which allowed us to analyze phosphoproteins in vivo and to evaluate their phosphorylation sites and phosphorylation levels. In this study, we have identified nearly 2500 phosphopeptides in barley grains when being exposed to water, and analyzed their differential regulations between the dormant and the AR states.

## 2. Results and Discussion

### 2.1. Phosphoproteomic Analysis of Imbibed FH and AR Grains

To understand the phosphosignaling pathways that operate during the imbibition of matured FH and AR grains, phosphoproteomic analysis was performed in this study. In our phosphoproteomic analysis, we have used barley half-grains in which husk-based dormancy is broken and only embryo-based dormancy is present. In addition, the embryo was dissected and used for phosphoproteomic analysis, to remove a large amount of storage proteins contained in the endosperm.

**Figure 1:**
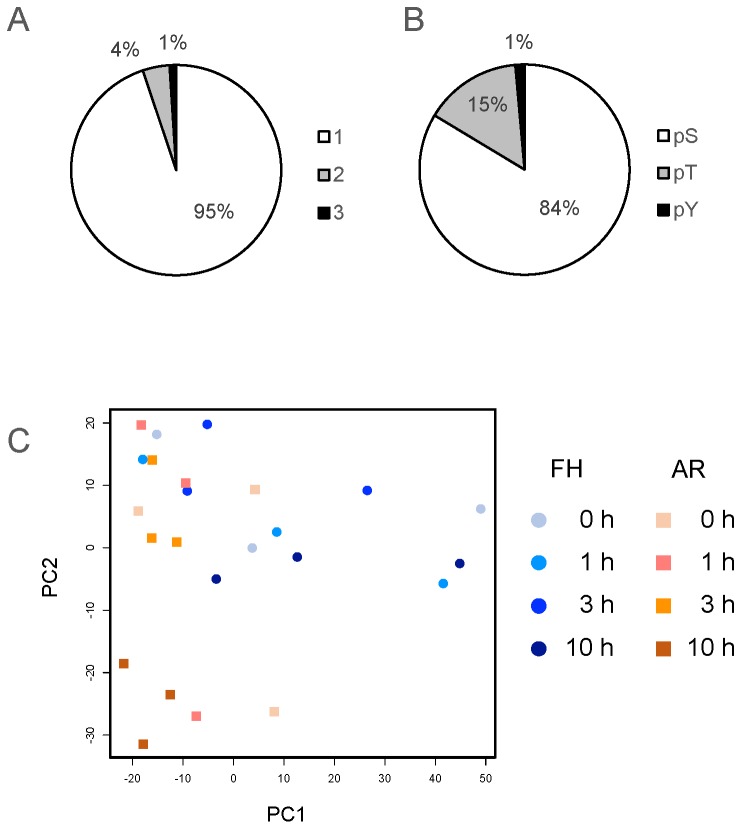
Summary of phosphoproteomic data. (**A**) Frequency of phosphorylated residues distributed in the phosphopeptides. Each number indicates the number of phosphorylation sites in phosphopeptides. (**B**) Distribution of phosphorylated residues in each phosphopeptide. pS, pT and pY showed phosphorylated serine, threonine and tyrosine, respectively. (**C**) Sample pattern recognition in principal component analysis. Blue circles and orange squares indicate FH and AR samples, respectively.

FH or AR half-grains were imbibed for 0, 1, 3 and 10 h, and then embryos were dissected under the microscope; and proteins were isolated from these tissues and used for phosphoproteomic analyses. LC-MS/MS analysis identified 2346 phosphopeptides and 2491 phosphorylation sites in FH and AR grains, respectively (Table S1). About 95% of these were singly phosphorylated peptides, and 5% of them were multiply phosphorylated (Figure 1A). The most prominent phosphorylated amino acid was phosphoserine (84%), followed by phosphothreonine (15%), while only 1% was phosphotyrosine (Figure 1B). Phosphoproteomic analyses in other plants, such as Arabidopsis, rice and *Physcomitrella patens*, found a similar distribution of phosphorylated residues [26,27,28,29,30,31].

Datasets from FH and AR samples were compared and phosphopeptide changes and phosphorylation levels were analyzed via principal component analysis (PCA) of the total identified phosphopeptide data (Figure 1C). FH samples showed a similar localization in the PC1–PC2 projection, while the 10 h sample appeared as the most different. The AR samples showed a very different distribution from FH samples after imbibition. While small differences between FH and AR were seen at 0 h and 1 h, the separation between them became very significant at 10 h. These results suggest that AR embryos experience larger phosphopeptide changes than FH embryos during imbibition, with the AR 10 h sample the most different from the set. This may reflect the deep physiological changes occurring during germination.

### 2.2. Classification of Phosphopeptides in Barley Grains

**Figure 2:**
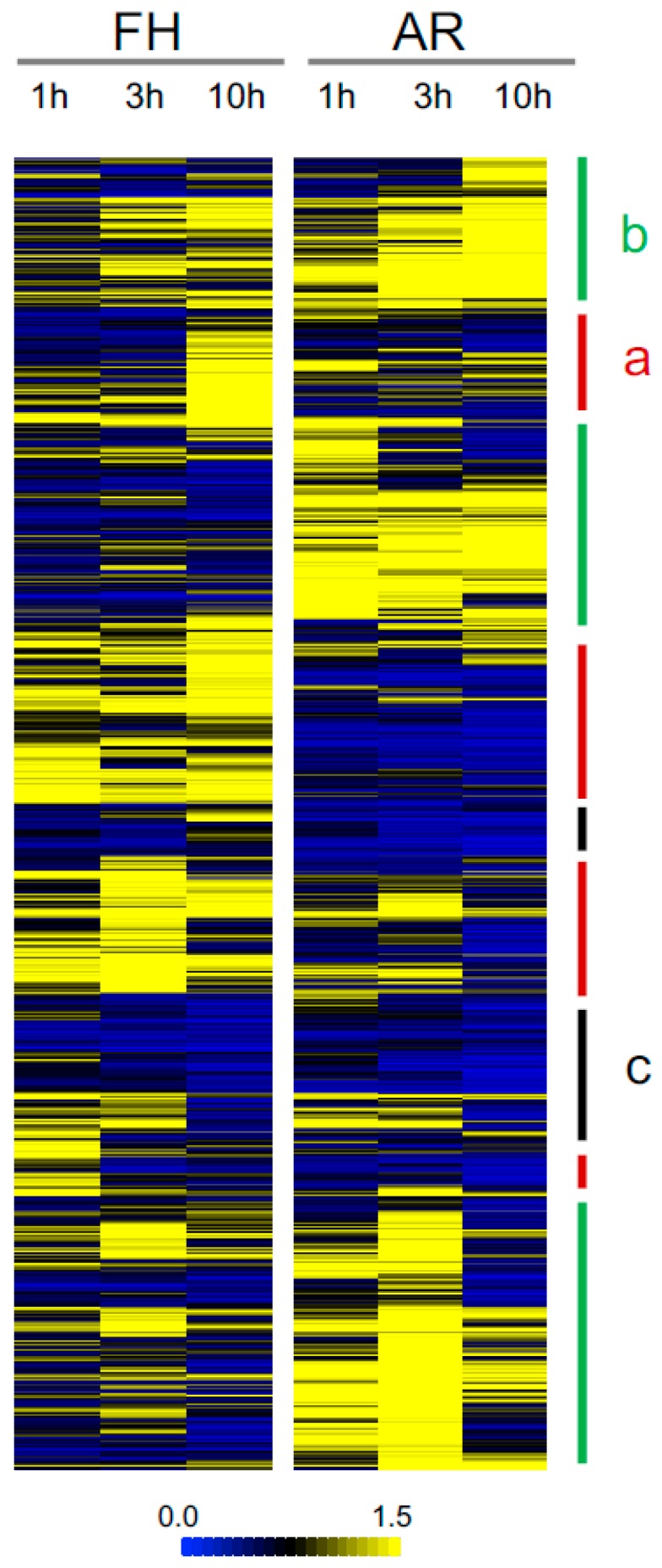
Comparative analysis of FH and AR barley grains. Quantitative data of each phosphopeptide in FH and AR grains was displayed as a heatmap. Phosphopeptides could be classified into clusters a, b and c based on their phosphorylation patterns. Cluster a (red) and cluster b (green) include phosphopeptides primarily showing large increases in FH and AR grains, respectively. The cluster c (black) includes phosphopeptides that showed similar tendencies in both samples.

To compare phosphoproteome between FH and AR grains, quantitative data of each phosphopeptide was used for a clustering analysis. Hierarchical clustering analysis showed that most phosphopeptides were upregulated in FH and AR grains, but some of them were differentially regulated in FH and AR grains (Figure 2). This analysis divided phosphopeptides into three clusters. The first and second clusters include phosphopeptides primarily showing large increases in either FH (cluster a) or AR grains (cluster b), respectively. The third cluster included phosphopeptides that showed similar tendencies in both samples (cluster c). Cluster b was the largest, and cluster c contained the fewest members in this analysis. AR samples showed a different tendency between 0 and 10 h in comparison with FH samples. This result was consistent with PCA, suggesting that AR grains change phosphorylation status more than FH during imbibition.

**Figure 3:**
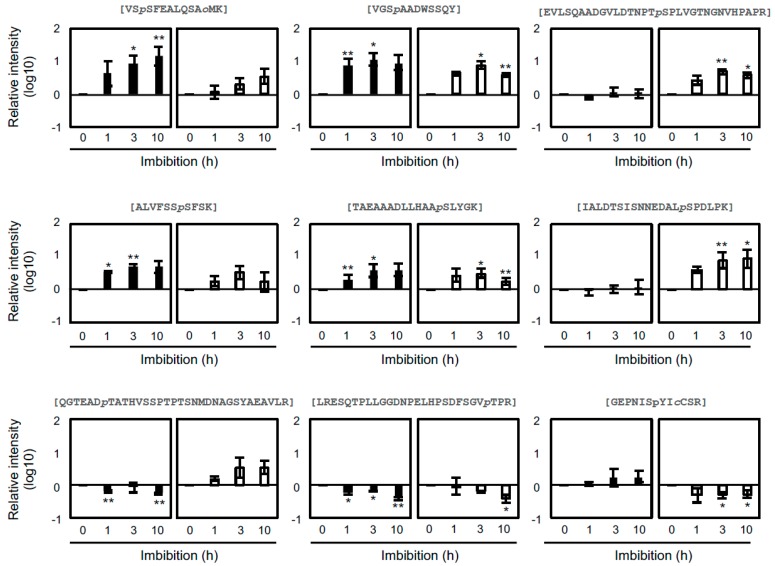
Examples of phosphopeptides in barley seeds. Quantitative data of each phosphopeptide was analyzed for FH (solid) and AR (empty) grains treated with imbibition. Bars indicates ± standard error (*n* = 3), and * and ** indicate *p*-values of <0.05 and <0.01, respectively.

To examine the most robust changes, we screened phosphopeptides that statistically increased or decreased in imbibition as compared to the 0 h for each seed stage. Of these, 98 and 199 phosphopeptides increased in FH and AR embryos, respectively (Table 1; Table S2), with only 28 of them (10.5%) being shared between the two sets. Conversely, 39 and 59 phosphopeptides decreased in FH and AR embryos, respectively (Table 1; Table S2). Interestingly, only one phosphopeptide was shared between these two sets. In accordance with Figure 2, the number phosphopeptides that increased was more than those that decreased. Examples of phosphopeptides with different patterns are shown in Figure 3. Some phosphopeptides showed significant changes specifically in FH and/or AR samples. Two of those phosphopeptides exhibited converse accumulation patterns in FH and AR embryos. For example, glycosyl hydrolase family protein was upregulated in the FH embryo and downregulated in the AR embryo.

### 2.3. Comparative Analysis of Phosphopeptides

**Figure 4:**
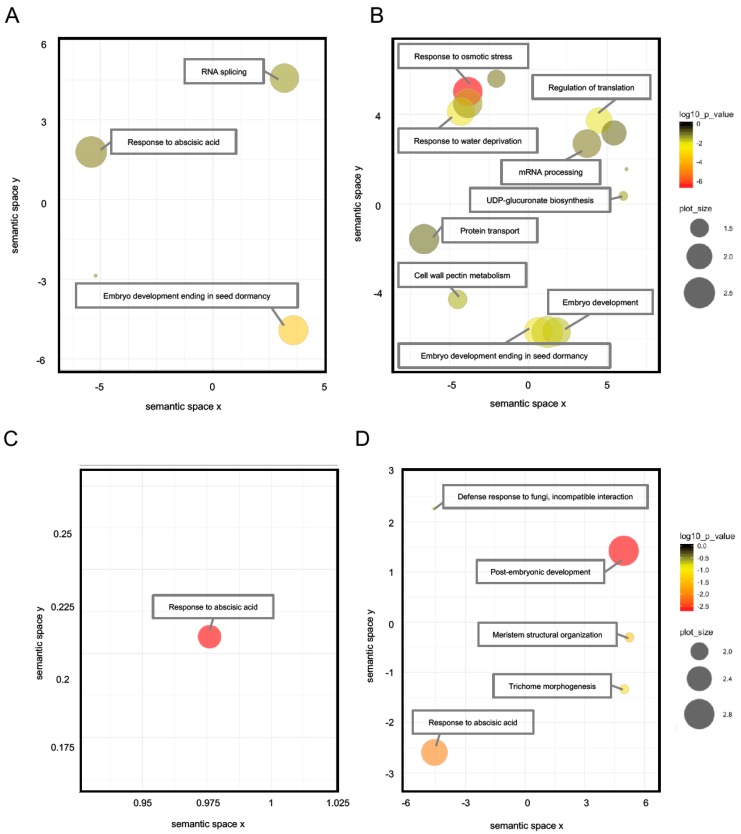
GO analysis of phosphopeptides in barley seeds. GO terms were evaluated by DAVID program and visualized with REViGO for phosphopeptides upregulated and downregulated under imbibition. Each circle color and size show *p*-value and frequency (%), respectively. Phosphopeptides used for this analysis included 70 and 171 phosphopeptides upregulated in FH (**A**) and AR (**B**) seeds, respectively; 38 and 58 phosphopeptides downregulated in FH (**C**) and AR (**D**) seeds, respectively.

Phosphopeptides were further analyzed to examine potential differences in biological processes between FH and AR embryos. First, barley genes were annotated using the Arabidopsis database (TAIR10); and gene ontology analyses of up- and downregulated phosphopeptides was performed. The 70 and 171 phosphopeptides found in Table 1 to be uniquely upregulated in FH only and AR only, respectively, were used for gene ontology (GO) analysis (Figure 4A,B; Table S3). Proteins upregulated in FH grains were enriched in GO categories related to “response to ABA”, “embryo development ending in seed dormancy” and “RNA splicing” (Figure 4A). Uniquely enriched in AR grains responses included “response to osmotic stress”, “embryo development ending in seed dormancy”, cell wall pectin metabolism”, “regulation of translation” and “mRNA processing” (Figure 4B). To compare FH with AR, “response to ABA” in FH was enriched; and enrichment of “embryo development ending in seed dormancy” was lower in FH than in AR. Especially the GO term of “cell wall pectin modification” was highlighted to associate with germination. During germination, embryo growth and cell wall degradation occur to be associated with physiological and physical dormancy, respectively [32,33,34,35,36,37]. This GO term indicates AR grains go toward radicle protrusion, germination.

From Table 1, the 38 and 58 downregulated phosphopeptides unique to each seed stage were used for GO analysis (Figure 4C,D). For FH grains, GO term was enriched for “response to ABA” (Figure 4C). GO terms of “response to ABA”, “post-embryonic development”, “meristem structural organization” and “trichome morphogenesis” were enriched in AR (Figure 4D). Among GO terms of AR grains, “post-embryonic development” was the most highlighted one. ABA-related phosphopeptides were significantly downregulated in both of grains. The phytohormone ABA plays an important role in response to environmental stress and dormancy [7,9,23,38]. Recently, it was reported that ABA responses involve activation of protein kinase SnRK2, and then activated SnRK2 phosphorylates downstream substrates, including bZIP transcription factors [22,39,40,41,42], to modulate their activity. The enrichment of the GO term “response to ABA” in phosphopeptides decreasing in AR is consistent with the decay of ABA signaling that would be associated with the rapid decline in dormancy in AR seeds after imbibition.

**Figure 5:**
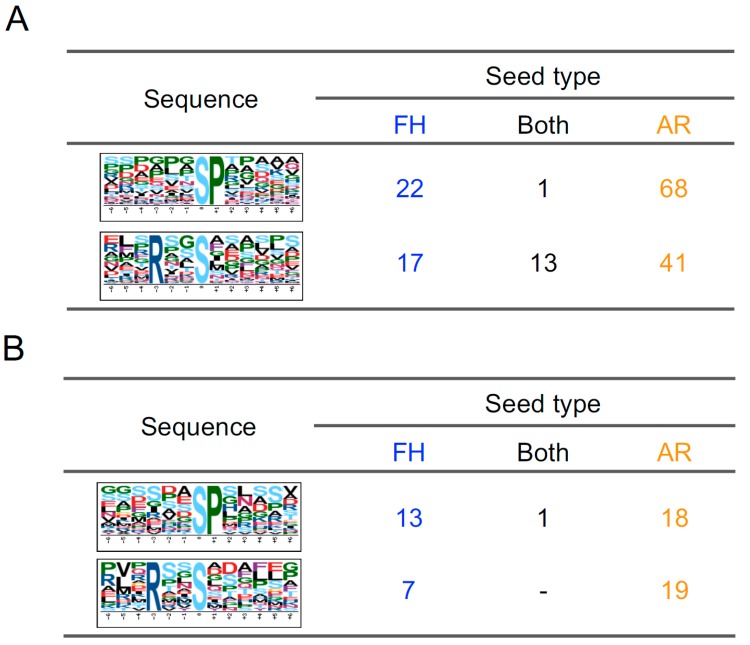
Motif analysis of phosphopeptides in barley grains. Motif analysis of 269 upregulated phosphopeptides (**A**) and 97 downregulated phosphopeptides (**B**) using the motif-x algorithm. Extracted phosphorylation motifs were counted in the data from FH or AR grains.

Phosphorylation motif analysis can indicate some kinases upstream of the differentially phosphorylated proteins, thus pointing to kinases that may be changing in activity during these processes [43,44]. In this study, two phosphorylation motifs, [-pS-P-] and [-R-x-x-pS-], were enriched in both up- and downregulated candidates (Figure 5; Table S4). [-pS/-P-] is a known mitogen-activated protein kinase (MAPK)- and cyclin-dependent kinase (CDK) target motif. SnRK2, calcium-dependent protein kinases (CDPK) and CBL-interacting protein kinases (CIPK) phosphorylate on [-R-x-x-pS-] motifs. Among 70 upregulated phosphopeptides in the FH sample, 22 (30.9%) and 17 (23.9%) include [-pS/T-P-] and [-R/K-x-x-pS/T-], respectively, while 68 [-pS/T-P-] (38.4%) and 41 [-R/K-x-x-pS/T-] (23.1%) were found in 171 upregulated phosphopeptides in the AR sample (Figure 5A).

Next, a motif analysis was performed for 97 phosphopeptides which were downregulated after imbibition. As well as upregulated phosphopeptides, [-R/K-x-x-pS/T-] and [-pS/T-P-] were identified in FH and AR grains (Figure 5B). Actually, it is difficult to understand why the same motifs were enriched in both upregulated and downregulated phosphopeptides. It may result from different protein kinases sharing the same target motifs as described above.

### 2.4. Differential Regulation Mechanisms of Seed Dormancy

Various approaches have been performed to investigate the differences between FH and AR grains in cereals [45,46,47,48,49]. A variety of different factors have been reported to contribute to the antagonistic regulation of dormancy and germination, including environmental responses, such as light and temperature, oxidation of proteins, and differential accumulation of transcripts (i.e., changes in gene expression) [50,51]. These previous studies indicated that ABA has an indispensable role in dormancy regulation. The perception of changes in ABA is primarily transmitted by three major components: ABA receptors (PYR/PYL/RCAR), protein phosphatases (PP2Cs) and protein kinases (SnRK2s) [39,40,41]. Both ABA content and signaling are important for controlling plant responses [45,52], and it can be difficult to separate both elements because of the complex feed-back regulation of the ABA synthesis pathway. Millar et al. reported that the ABA content in dry seeds is similar between FH and AR samples, and that only after imbibition a difference occurs due to the AR seeds being unable to maintain high ABA levels [45]. This study and others would suggest that ABA signaling changes during after-ripening are more critical than content changes for the regulation of dormancy and germination. In agreement with this result, our GO analysis showed a set of phosphopeptides, of which responses to imbibition are related to “response to ABA”. In FH, up- and downregulated phosphopeptides contained “response to ABA”. On the other hand, “response to ABA” was strongly enriched in downregulation in comparison with upregulation in AR. These results suggest ABA signaling is active in FH, but it is impaired by imbibition in AR. Additionally, most of phosphopeptides containing [-R/K-x-x-pS/T-] decreased their phosphorylation level with imbibition in AR. This indicates the activities of SnRK2 and/or CDPK, which are involved in ABA signaling and target [-R/K-x-x-pS/T-], are impaired during imbibition. These results consistently imply ABA signaling is attenuated in the AR grain compared to that in the FH grain. Although the ABA contents of FH and AR grains decrease, ABA signaling is activated or repressed in FH and AR grains, respectively. It is still unclear how a decay in ABA signaling during imbibition is induced during after-ripening.

The influence of DELAY OF GERMINATION1 (DOG1) is one possible to alter ABA signaling between FH and AR grains. DOG1 is expressed in fresh/dormant and also in AR Arabidopsis seeds, but DOG1 proteins are less abundant or downregulated in AR seeds [53,54]. Interestingly, recent studies have reported that DOG1 interacts with AHG1 and AHG3, one of PP2Cs in clade A, and is able to repress its function directly [55,56]. This inhibition possibly induces the activation of SnRK2 in FH grains, but not in AR grains.

### 2.5. The Role of Abscisic Acid in Seed Dormancy during Water Imbibition

We identified the ortholog of AREB3 and other ABA-responsive proteins were downregulated in AR grains during water imbibition. AREB3 belongs to group A bZIP transcription factors, which are responsible for ABA-responsive element (ABRE; PyACGTGG/TC)-dependent gene expression [57,58]. These factors are divided into two subclasses: ABRE-binding protein (AREB)/ABRE-binding factor (ABF) subfamily having a role in the vegetative tissue and ABA-INSENSITIVE 5 (ABI5)/Dc3 promoter-binding factor (DPBF) subfamily working in the seed [59,60]. Group A bZIP transcription factors are phosphorylated by SnRK2 and activate the gene expression in response to ABA [27,61,62,63]. bZIP transcription factors could be regulated by the ABA content. Millar et al. revealed ABA content decreased sharply after water imbibition in AR grains in comparison with FH (dormant) grains of barley [45]. Low ABA concentration cannot influence ABA signaling enough. Additionally, bZIP transcription factors could be controlled by dephosphorylation. Group A bZIP transcription factors were indicated to be dephosphorylated by PP2C [64,65]. In low ABA conditions, released PP2C possibly dephosphorylates SnRK2 and bZIP transcription factors to repress ABA signaling. Another possibility is that protein degradation negatively regulates group A bZIP transcription factors. ABI5 is known to be degraded by KEEP ON GOING (KEG) and CUL4/DDB1 E3 Ligase [66,67,68,69,70]. It is presumed that activated E3 ubiquitin ligases degrade bZIP transcription factors.

Taken together, this study performed phosphoproteomic analyses of FH and AR embryos in barley during imbibition and demonstrated differential phosphosignals in FH and AR barley grains. We have identified numerous phosphopeptides and 365 of them significantly altered phosphorylation levels during imbibition. These phosphopeptides are possibly involved in control of dormancy and germination, and some of them could be involved in the regulation of ABA signaling. Further studies will be required for understanding the role of these responsive phosphoproteins and the upstream elements that regulate the activity of various protein kinases during after-ripening.

## 3. Materials and Methods

### 3.1. Plant Material and Growth Condition

Barley (*Hordeum vulgare* cv. Golden Promise) plants were grown in a phytotron glasshouse (CSIRO, Canberra, Australia) under sunlight and temperature set at 17/9 °C day/night [71]. Grains were harvested at physiological maturity and half of the harvest was stored at −20 °C to preserve a dormancy level as FH. The other half was after-ripened at 37 °C for six months to impair dormancy and then stored at −20 °C as well (AR).

### 3.2. Phosphoproteomic Analysis

Twenty half-cut grains were prepared and set on filter paper (9 cm in diameter, Whatman #1, GE Healthcare, Chicago, IL, USA) in plastic petri dishes. After adding 5 mL double-distilled H_2_O, dishes were sealed with a Parafilm and covered by aluminum foil, and then incubated at 20 °C for each time course, 1 h, 3 h and 10 h.

Following imbibition, embryos were dissected from barley half grains and stored at −80 °C as previously described [47,72]. Fifteen embryos were grounded by using TissueLyser II (QIAGEN, Germantown, MD, USA), and samples were resuspended in 1 mL of protein extraction buffer containing 10 mM Tris-HCl (pH 9.0), 8 M Urea, 2% Phosphatase Inhibitor Cocktail II (Sigma, St. Louis, MO, USA) and 2% Phosphatase Inhibitor Cocktail III (Sigma, St. Louis, MO, USA). After centrifugation at 17,400 g at 4 °C for 10 min, supernatants were collected as crude extracts, and protein concentrations were measured by BCA Protein Kit (Thermo Scientific, San Jose, CA, USA).

The phosphoproteomic analyses were performed as previously described [27,30,59,60] with minor modifications. Aliquots of 400 µg total protein were reduced with 10 mM DTT for 30 min, and alkylated with 50 mM iodoacetamide for 20 min in the dark, and then with Lys-C (WAKO, Osaka, Japan; 1:200, *w*/*w*) for 3 h. After 4-fold dilution with NH_4_HCO_3_, proteins were digested with trypsin (Promega, Madison, WI, USA; 1:100, *w*/*w*) overnight at room temperature.

After enzymatic digestion, an equivalent volume of 2% trifluoroacetic acid (TFA) was added to the digested samples, and then they were desalted using SDB-XC Empore disk membranes (3M, St. Paul, MN, USA) as described previously [73]. To enrich phosphopeptides, the hydroxyl acid-modified metal oxide chromatography (HAMMOC) method was performed [74]. Custom-made metal oxide chromatography (MOC) tips made with C8-StageTips and 3 mg of bulk titania beads (particle size, 10 µm; GL science, Torrance, CA, USA) were used in this study. The concentrated phosphopeptide sample was desalted with a C18-SDC and C18-GC column (GL science, Torrance, CA, USA). Each column was washed with solution A (80% acetonitrile and 0.1% TFA) and 0.1 % TFA by using centrifugation at 700 g for 2 min at room temperature. Samples were loaded on each column and centrifuged at 700 g at room temperature. After being washed with 0.1% TFA, phosphopeptides were eluted with solution A by using centrifugation at 700 g for 2 min. Samples were dried in a vacuum evaporator (Tomy, Tokyo, Japan), and diluted with 10 µL of 0.1% formic acid (FA).

Cleaned-up samples were analyzed with TripleTOF 5600 system (AB-SCIEX, Framingham, MA, USA) equipped with Autosampler-2 1D plus (Eksigent, Framingham, MA, USA) and NanoLC Ultra (Eksigent, Framingham, MA, USA) using MonoCap C18 High Resolution 2000 column (GL science, Torrance, CA, USA) and PicoTip emitter SilicaTip (New Objective Inc., Woburn, MA, USA). Peptides were eluted at 500 nL min^−1^ with a four-step gradient, 0.5% acetic acid: 0.5% and 80% acetic acid = 98:2 (0 min), 60:40 (300 min), 10:90 (20 min) and 98:2 (40 min). The eluate was sprayed into mass spectrometer by electrospray ionization (ESI). The mass spectrometry (MS) scan range was 400–1250 m/z and the MS/MS scan range was 100–1600 m/z.

### 3.3. Phosphopeptide Identification and Quantification

Peak lists were generated using Protein pilot version 5.0.0.4769 (AB-SCIEX, Framingham, MA, USA). Raw spectrum files were matched with the barley gene database published on 23 March 2012 (Plant Genome and Systems Biology; https://www.helmholtz-muenchen.de/pgsb) using Mascot version 2.4.0 (Matrix Science, London, UK). Search settings were applied: a precursor mass tolerance of 3 ppm, a fragment ion mass tolerance of 0.8 Da, and cut-off value of 0.95, allowing for up to two miss cleavages, with the enzyme designated as trypsin. A fixed modification of carbamidomethylation of cysteine and variable modifications of oxidation of methionine and phosphorylation of serine, threonine and tyrosine were used. All raw data files were deposited in the Japan Proteome Standard Repository/Database (jPOST; JPST000502, Kyoto, Japan).

Skyline software version 4.2 (https://skyline.ms/project/home/software/Skyline/begin.view) was used for phosphopeptide quantification from peak areas [75]. The search settings were the same as described for Mascot. The maximum false discovery rate (FDR) thresholds for protein was set to 5%. In addition, the site localization probability threshold was specified as >0.75. Fold changes were calculated using quantitative values. For each time point, three biological replicates were analyzed and the significance of time-dependent changes was determined by Student’s *t* test (*p* < 0.05).

### 3.4. Data Analysis

Each phosphoproteomic sample including FH and AR grains was compared by PCA [76,77,78]. Samples were plotted with principal component 1(PC1) and PC2. Hierarchical clustering analysis was performed on phosphorylation intensity using Multi Experimental Viewer (MeV, Boston, MA, USA). Pearson correlation and average linkage clustering were applied for settings. Gene Ontology (GO) analysis was performed with DAVID (https://david.ncifcrf.gov) and REViGO (http://revigo.irb.hr). Annotated data with Arabidopsis by BLAST (https://blast.ncbi.nlm.nih.gov/Blast.cgi) was loaded to DAVID, and background database was set as the TAIR 10 Arabidopsis dataset. Outputted GO terms in DAVID were visualized with REViGO. Settings used for REViGO were: medium (0.7) similarity, UniProt Arabidopsis database (https://www.uniprot.org) and simRel semantic measure. Phosphorylation motifs were predicted by the motif-x program (http://motif-x.med.harvard.edu) [79]. For motif analysis, 13 amino acids around phosphorylated residues were extracted from identified phosphopeptide sequences, and submitted to motif-x, setting an occurrence to 20 and significance to 0.01. Barley expressed sequence tag (EST) data was submitted to the local BLAST program against the Arabidopsis dataset (TAIR10) to make a list of orthologues [80].

## 4. Conclusions

To understand the phosphosignaling that take place during the after-ripening of barley grains and that produce a decay in dormancy, phosphoproteomic profiles were obtained from FH and AR embryos during imbibition. As a result, 2,346 phosphopeptides were identified, with 365 of them responded to imbibition. Our data indicate that multiple protein kinases, such as SnRK2, CDPK, CIPK, or MAPK, can actively participate in the differential phosphorylation of peptides in barley FH or AR grains, and point to some key kinases that could be manipulated for regulating germination in cereals.

## Figures and Tables

**Table 1:** The numbers of up- and downregulated phosphopeptides in barley grains during imbibition.

Response	Freshly Harvested	Overlap	After-Ripened	Total
**Upregulated**	98	28	199	269
**Downregulated**	39	1	59	97

Comparative analysis selected phosphopeptides which were upregulated or downregulated in response to imbibition in FH and AR grains. Each phosphopeptide was statistically tested by Student’s *t*-test (*p*-value < 0.05).

## Supplementary Materials

Supplementary materials can be found at http://www.mdpi.com/1422-0067/20/2/451/s1.

## Abbreviations

FH	freshly harvested
AR	after-ripened
LC-MS/MS	liquid chromatography–mass spectrometry/mass spectrometry
