# Supplementary material for: Comparative Phosphoproteomic Analysis of Barley Embryos with Different Dormancy during Imbibition
Source: Int J Mol Sci. 2019 Jan 21;20(2):451. doi: 10.3390/ijms20020451 (PMC6359383; doi:10.3390/ijms20020451)
Supplement: Supplementary file 1 [file ijms-20-00451-s001.zip › ijms-418799 sp for proof 1.18/New Microsoft Word Document.pdf]

Supplementary materials

Table S1. List of phosphopeptides in barley grains.
Freshly harvested or after-ripened barley grains were exposed to water for 0, 1, 3, and 10 h and enriched phosphopeptides were analyzed with a liquid chromatography–tandem mass spectrometry (LC-MS/MS) system. Tables consist of relative peak area of each phosphopeptides with annotated information (sequence, modification, or protein description, etc.), for three replicates.

Table S2. List of responded phosphopeptides during imbibition.
Quantitative data of phosphopeptides were derived from FH and AR grains treated with imbibition. This list shows phosphopeptides which were upregulated or downregulated in FH or AR grains with p-value less than 0.05 for at least one of the time points analyzed.

Table S3. List of GO terms for phosphopeptides in barley grains.
The GO terms were enriched phosphopeptides, which were upregulated or downregulated in FH or AR grains, using DAVID program.

Table S4. Classification of phosphopeptides by motif groups.
Phosphorylation motifs were analyzed for phosphopeptides in barley grains using Motif-x program. Each motif group was defined as follows: Group 1, [-R/K-x-x-pS/pT-] and Group 2, [-pS/pT-P-].
